# Enhancement of Rubella Virus Infection in Immortalized Human First-Trimester Trophoblasts Under Low-Glucose Stress Conditions

**DOI:** 10.3389/fmicb.2022.904189

**Published:** 2022-07-08

**Authors:** Quang Duy Trinh, Kazuhide Takada, Ngan Thi Kim Pham, Chika Takano, Takahiro Namiki, Ryo Ikuta, Shingo Hayashida, Shoko Okitsu, Hiroshi Ushijima, Shihoko Komine-Aizawa, Satoshi Hayakawa

**Affiliations:** ^1^Division of Microbiology, Department of Pathology and Microbiology, Nihon University School of Medicine, Tokyo, Japan; ^2^Nihon University School of Medicine, Tokyo, Japan; ^3^Department of Pediatric Surgery, Nihon University School of Medicine, Tokyo, Japan

**Keywords:** rubella, trophoblasts, pregnancy, infection, low glucose, endoplasmic reticulum stress, congenital rubella syndrome

## Abstract

Rubella virus (RuV) infections in pregnant women, especially first-trimester infections, can lead to congenital rubella syndrome (CRS). However, the mechanisms of fetal RuV infection are not completely understood, and it is not observed in every pregnant woman infected with RuV. As gestational diabetes mellitus is a risk factor for congenital viral infections, we investigated the possible roles of hypoglycemia-related endoplasmic reticulum (ER) stress as a key factor for vertical RuV infection using immortalized human first-trimester trophoblasts. Low-glucose stress was induced prior to RuV infection by culturing HTR-8/SVneo and Swan.71 cells in low-glucose (LG) medium for 24 h or high-glucose medium for 6 h and then LG medium for an additional 18 h. Clinically isolated RuV was inoculated at a multiplicity of infection of 5 to 10. The intracellular localization of the RuV capsid protein was investigated 24 to 48 h post-infection (pi) with flow cytometry (FCM) analysis and fluorescence microscopy. Viral progeny production was monitored by FCM analysis. Increases in RuV infection in LG-induced ER-stressed trophoblasts were observed. No significant increase in apoptosis of RuV-infected cells was noted at days 2 and 5 pi, and substantial viral progeny production was observed until day 5 pi. An approximate fivefold increase in viral binding was noted for the LG-stressed cells. Although the detailed mechanisms underlying viral entry into LG-stressed cells are not known and require further investigation, these findings suggest that a certain degree of LG stress in early pregnancy may facilitate infection and cause CRS.

## Introduction

Rubella virus (RuV) belongs to the family *Matonaviridae* and genus *Rubivirus* (Chen et al., 2018). RuV causes a mild, rash-producing, febrile illness in children, and it is well known for causing stillbirth, premature birth, or congenital defects called congenital rubella syndrome (CRS) in pregnant women, particularly during the first trimester. CRS occurs after the transplacental transmission of RuV during the first 8 weeks of gestation in up to 90% of cases and during the second trimester in 25–35% (Hobman, 2013). Although most studies attribute fetal susceptibility to RuV-related teratogenesis in the first trimester of pregnancy to the critical periods of major organogenesis, the mechanisms involved in *in utero* transmission during these critical periods are not well established.

During the first trimester of pregnancy, the primary placental cells derived from the trophectoderm of the blastocyst, cytotrophoblasts (CTBs), differentiate into extravillous trophoblasts (EVTs), which invade uterine tissue and replace uterine spiral arterial walls (Silva and Serakides, 2016; Yoshida et al., 2021). This induction of trophoblast differentiation is promoted by low oxygen concentrations during placental formation in early pregnancy, which is also associated with enhanced endoplasmic reticulum (ER) stress (Chang et al., 2018).

The ER is a large cellular organelle that plays an essential role in protein synthesis and maturation. Excessive accumulation of unfolded or misfolded proteins in the lumen of the ER during conditions of hypoxemia, oxidative stress, impaired Ca^2+^ homeostasis, and glucose deprivation causes ER stress (Ni and Lee, 2007; Schwarz and Blower, 2016; Bastida-Ruiz et al., 2017; Burton et al., 2017; Guzel et al., 2017). Increased ER stress occurs naturally in the first trimester of pregnancy and in women with gestational diabetes mellitus (GDM), pregnancies are complicated with fetal growth restriction, preeclampsia, and during labor (Burton and Yung, 2011; Lian et al., 2011; Veerbeek et al., 2015; Yung et al., 2016). Of importance, patients with GDM are more suspicious for viral infections, including COVID-19 (Mamun and Khan, 2021).

We recently discovered that RuV has low infectivity to the first-trimester trophoblast cell lines HTR-8/SVneo and Swan.71 *in vitro*, suggesting that there might be other factors affecting the infectivity of RuV to these cells (Pham et al., 2022). Searching for such possible factors led us to hypothesize that ER stress may play a role in RuV infection in early pregnancy.

To partially answer the above question, the present study investigated the influence of low-glucose-induced ER stress on RuV infection in the immortalized human first-trimester trophoblast cells HTR-8/SVneo and Swan.71. By culturing these trophoblast cells in low-glucose (LG) medium for 24 h or high-glucose (HG) medium for 6 h and then LG medium for an additional 18 h to induce LG stress prior to RuV infection *in vitro*, we found that there was an enhancement of susceptibility to RuV in the treated trophoblast cells.

## Materials and Methods

### Cell Culture and Virus

HTR-8/SVneo cells (originally obtained from human first-trimester placentas and immortalized *via* transfection with a cDNA construct encoding simian virus 40 large T antigen) and Swan.71 cells (Sw.71, derived from the telomerase-mediated transformation of a 7-week cytotrophoblast isolate described; Graham et al., 1993; Straszewski-Chavez et al., 2009) were kindly provided by Dr. Gil Mor (Wayne State University, Detroit, MI, United States) and were cultured in RPMI 1640 medium (Gibco-Invitrogen, Tokyo, Japan) supplemented with 10% fetal bovine serum (FBS), 10 mM HEPES (Invitrogen), 0.1 mM nonessential amino acids (Invitrogen), 1 mM sodium pyruvate (Invitrogen), and 100 units/ml penicillin–streptomycin (complete medium). Vero cells were purchased from the Japanese Collection of Research Bioresources Cell Bank and cultured in Dulbecco’s modified Eagle’s medium (DMEM; Gibco-Invitrogen, Tokyo, Japan) supplemented with 10% FBS and 100 units/ml penicillin–streptomycin. All cells were cultured in monolayers at 37°C in a humidified 5% CO_2_ incubator.

The clinical RuV strain (3-B1-RK13) was transferred from Kitasato University School of Medicine (Tokyo, Japan). The viral stock solution was prepared by propagating the virus in Vero cells and concentrating the viral particles *via* ultracentrifugation at 52,000 × g for 90 min in a Himac CS100GX microultracentrifuge with an S50A rotor (Hitachi Koki Co., Ltd., Ibaraki, Japan). Viral titers were estimated with the TCID50 method or flow cytometry (FCM) analysis, as described previously (Grigorov et al., 2011; Pham et al., 2021).

### Experimental Low-Glucose-Induced ER Stress Conditions in Trophoblast Cells

Trophoblast cells were seeded in 6-well plates (10^5^ cells/well), 12-well plates (5 × 10^4^ cells/well), 24-well plates (2.5 × 10^4^ cells/well), or 96-well plates (5 × 10^3^ cells/well) in complete medium and cultured for 2 days prior to experimentation. For the low-glucose stress inductions, the cells were cultured in serum-free RPMI containing 0.1% bovine serum albumin (BSA) and LG (0.5 mM glucose) for 24 h or HG (25 mM glucose) for 6 h followed by LG for 18 h (HG-LG, 24 h in total) to mimic the placental conditions in gestational diabetes with hypoglycemia (Khunti et al., 2016; Sarina Li et al., 2019). The cells cultured in serum-free normal RPMI containing 11 mM glucose (SF medium) were used as a control for the LG stress treatment. For comparative references, the cells were also cultured in the complete RPMI medium (FBS for abbreviation) or SF medium containing the ER stress activator thapsigargin (Tg, 25 nM) for 24 h.

### Cell Viability Assay

Trophoblast cells were cultured in a 96-well plate and subjected to LG treatments, as described above. Cell viability was measured using a Cell Counting Kit-8 (Dojindo Laboratories, Kumamoto, Japan) according to the manufacturer’s instructions. The cell density in each well was measured at 450 nm using a microplate reader (iMark Microplate Absorbance Reader, Bio-Rad, Hercules, CA, United States).

### Western Blotting

Cells grown in 6-well plates were subjected to the LG treatments, as described above. The supernatant was removed 24 h posttreatment, and the cells were washed and lysed in 70 μl of cell lysis buffer (Cell Signaling Technology, Danvers, MA, United States). The protein concentrations in the lysates were quantified using a DC Protein Assay (Bio–Rad Laboratories, Inc., Hercules, CA, United States). Cell lysates were loaded onto a NuPAGE 4–12% Bis-Tris protein gel (Invitrogen) and separated by electrophoresis. The separated proteins were transferred to polyvinylidene fluoride membranes (Invitrogen), and nonspecific binding sites were blocked using 1% BSA in phosphate-buffered saline (PBS) containing 0.05% Tween 20. Membranes were incubated with a primary rabbit polyclonal anti-GRP78 antibody (Abcam, Cambridge, United Kingdom), a mouse monoclonal anti-CHOP antibody, or a rabbit α-tubulin antibody (Cell Signaling Technology) at 4°C overnight. Membranes were incubated with a horseradish peroxidase-conjugated secondary antibody (Cell Signaling Technology) for 30 min at room temperature (RT) and visualized with a luminescent image analyzer (Image Reader LAS-4000 Mini, Fujifilm, Tokyo, Japan). After visualization, antibodies attached to the membranes were removed using Restore PLUS Western Blot Stripping Buffer (Thermo Fisher Scientific, MA, United States), and the membranes were reused to investigate the expression of other proteins.

### Viral Infection

Cells were cultured in 96-well plates (for FCM analysis) or 6-well plates with glass coverslips for immunofluorescence (IF) assays, washed with SF medium 24 h posttreatment, and incubated with the virus at multiplicities of infection (MOIs) of 5 to 10 for a total of 3 h in a 35°C, humidified 5% CO_2_ incubator with gentle shaking every 10 ~ 15 min during the first hour. The supernatant was removed, the cells were washed, and the medium was replaced with a fresh medium containing 2% FBS. The percentage of cells infected with the virus was determined 24 to 48 h post-infection (hpi) by FCM analysis and IF assays at 48 hpi. Negative control cells (mock-infected, infected with heat-inactivated RuV, or not exposed to the primary antibody during the staining procedures), as well as positive controls using RuV-infected Vero (or A549) cells, were also performed in parallel for comparison. Supernatants from day 2 pi were collected daily and replaced with the fresh medium until day 7 pi to monitor viral progeny production by FCM analysis.

To determine the infectivity of the supernatants, a 30 μl volume of serial 3-fold dilutions of the supernatants was used to infect (in duplicate) freshly seeded Vero cells in a 96-well plate (4 × 104 cells/well). Medium containing 2% FBS and NH4Cl was added 6 hpi to prevent a second round of infection (final concentration of NH4Cl, 20 mM). The cells were collected 24 hpi and subjected to intracellular staining of RuV capsid protein. Viral titers [in infectious units (IUs)] were determined by FCM analysis. The viral titer in a sample was calculated as the average of 3 titers measured in 3 consecutive wells with a percentage of RuV-infected cells lower than 40% and higher than 0.3%, as described previously (Grigorov et al., 2011; Pham et al., 2021).

### Viral Binding Assay

The cells were seeded onto 12-, 24-, or 96-well plates and then subjected to the LG treatments, as described above. The cells were washed once with ice-cold PBS, and viral binding buffer (1% BSA in PBS containing 0.1% sodium azide) was added for incubation on ice for 10 min. The cells were inoculated with RuV on ice for 1 h with gentle shaking every 10–15 min. The cells were gently washed three times with ice-cold PBS, collected *via* trypsinization (for 96-well plates), subjected to surface staining procedures for RuV, and then subjected to FCM analysis. In other experiments, the cells were subjected to RNA extraction (for 24-well plates) and then real-time PCR or lysate collection (for 12-well plates) for Western blot analysis.

### Immunofluorescence Assay

Cells cultured on glass coverslips in six-well plates were subjected to LG treatments and then incubated with RuV, as described above. The supernatant was removed 48 hpi, and the cells were fixed with cold methanol for 5 min, washed with PBS, and incubated with the mouse monoclonal anti-RuV capsid antibody (ab34749, Abcam) for 1 h at RT. Separate negative controls subjected to mock treatment, heat-inactivated RuV inoculation, and staining with normal mouse serum were established. The cells were washed with PBS and incubated with a goat anti-rabbit IgG H&L (Alexa Fluor 488) secondary antibody solution (ab150081, Abcam) for 30 min at RT. The samples were counterstained with 4′,6-diamidino-2-phenylindole dihydrochloride (DAPI; Lonza, Walkersville, MD, United States) for nuclear staining. After washing, the coverslips were mounted with VECTASHIELD Mounting Medium (Vector Labs, Burlingame, CA, United States), and fluorescence images were acquired using a fluorescence microscope (FLoid Cell Imaging Station; Life Technologies, CA, United States).

### FCM Analysis

For determination of the percentages of RuV-infected cells by FCM analysis, the cells were collected *via* trypsinization or detachment medium [RPMI containing 2.9 mM EDTA, 2% FBS, Live/Dead Staining Solution (Live/Dead Fixable Near-IR Dead Cell Stain Kit, Thermo Fisher Scientific, MA, United States)]. The cells were washed with staining buffer (STB, cold PBS containing 5% FBS and 2 mM EDTA) and fixed and permeabilized using a BD Cytofix/Cytoperm Fixation/Permeabilization Solution Kit (BD Biosciences, San Diego, United States). Intracellular staining was performed with a mouse monoclonal anti-RuV capsid antibody (ab34749, Abcam) for 30 min at RT. The cells were washed and incubated with a goat anti-mouse IgG H&L (Alexa Fluor^®^ 647) secondary antibody (ab150115, Abcam) solution for 30 min at RT. The cells were subjected to FCM analysis after washing and fixation. For each sample, at least 5,000 gated events were collected and analyzed on a BD FACSVerse cytometer using BD FACSuite software (version 1.2; BD Biosciences). Separate negative control groups without viral inoculation or incubated with heat-inactivated RuV were also established. For FCM analysis after the viral binding assay, cell surface staining procedures for RuV were performed on ice after trypsinization and STB washing using the same primary and secondary antibodies described above, with 1 h for each incubation.

### RNA Extraction and RT-PCR

HTR-8/SVneo and Swan.71 cells were cultured in 24-well plates and subjected to LG culture conditions without or with RuV infection (in a viral binding assay), as described above. Total mRNA was extracted using TRIzol reagent (Life Technologies, Tokyo, Japan), and contaminated genomic DNA was removed by treatment with DNAse I (TaKaRa Bio, Inc., Otsu, Japan). Real-time RT-PCR was performed using a One-Step TB Green Prime-Script PLUS RT-PCR Kit (Perfect Real Time; TaKaRa Bio) in a QuantStudio 3 Real-Time PCR System (Applied Biosystems, MA, United States). The following primer sequences were used: GRP78/BiP, sense, 5′-TGT TCA ACC AAT TAT CAG CAA ACT C-3′ and antisense, 5′-TTC TGC TGT ATC CTC TTC ACC AGT-3′; CHOP, sense, 5′-AGA ACC AGG AAA CGG AAA CAG A-3′ and antisense, 5′-TCT CCT TCA TGC GCT GCT TT-3′; RuV, sense, 5′-CCA CTG AGA CCG GCT GCG A-3′; antisense, 5′-GCC TCG GGG AGG AAG ATG AC-3′; and peptidylprolyl isomerase A (PPIA), sense, 5′-ATG CTG GAC CCA ACA CAA AT-3′ and antisense, 5′-TCT TTC ACT TTG CCA AAC ACC-3′. The results were analyzed using the delta–delta Ct method.

### Apoptosis Assay

Tests were performed on the studied trophoblast cells grown in 96-well plates using an ApoStrand ELISA apoptosis detection kit (BIOMOL, Plymouth Meeting, PA, United States). This detection system uses monoclonal antibodies to single-stranded DNA (ssDNA), which is present in apoptotic, but not necrotic, cells or cells with DNA breaks in the absence of apoptosis. The cells were seeded at a density of 5 × 10^3^ cells/well and cultured for 2 days prior to the LG treatments and RuV infection, as described above. ELISA was performed for apoptotic measurement at day 2 and day 5 pi. Briefly, the cells were fixed for 30 min with the kit fixative as indicated by the manufacturer and dried at 56°C for 20 min. Formamide was added to the cells, and the cells were heated at 56°C for 30 min to denature the DNA in apoptotic cells. After blocking, the cells were incubated with an antibody mixture for 30 min, washed, and incubated with 100 μl of peroxidase substrate for 45 min. The absorbance was read at 405 nm using an ELISA plate reader. Negative controls without viral inoculation and positive controls (provided in the kit) were also included.

### Statistical Analysis

Analysis of variance was used to analyze the results. A value of *p* < 0.05 obtained using the Tukey–Kramer test and Statcel 4 software (OMS Publishing, Inc., Tokorozawa, Saitama, Japan) was considered significant. Data were presented as the mean ± SEM.

## Results

### Upregulation of GRP78 and CHOP Under Low-Glucose Stress Conditions

**Figure 1:**
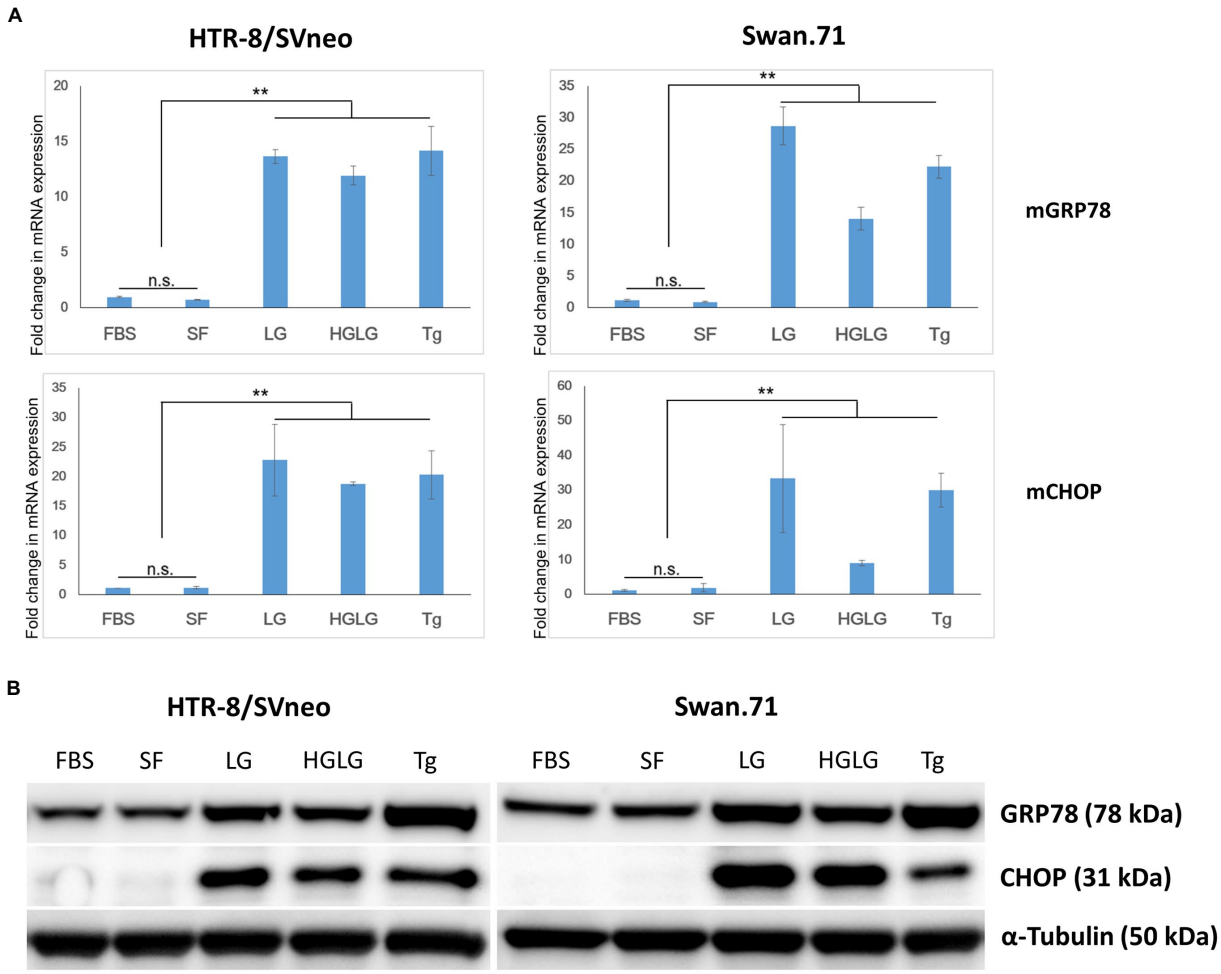
Expression changes in ER stress-related markers in first-trimester trophoblast cells under low-glucose-induced ER stress conditions. Trophoblast cells were cultured in 6-well plates (for WB) or 24-well plates (for RNA extraction and PCR) in complete medium for 2 days before culture in LG (low glucose, 0.5 mM) medium for 24 h, HG (high glucose, 25 mM) medium for 6 h, and then LG medium for an additional 18 h. The cells cultured in SF medium (serum-free, RPMI/0.1% BSA) were used as a negative control. The cells cultured in complete medium (FBS) or in SF medium containing 25 nM thapsigargin (Tg) for 24 h were used as comparative references. **(A)** Enhancement of GRP78 and CHOP mRNA expression. The results are representative of three independent experiments. ^**^p < 0.01. **(B)** Increased expression of the ER stress-related markers GRP78 and CHOP by Western blotting.

In some experiments, the LG treatment showed slightly lower cell densities, approximately 85 ~ 87% of the control group (the cells cultured in SF RPMI medium, data not shown) as measured by a Cell Counting Kit-8 assay. Real-time PCR demonstrated increased induction of the ER-stress-response gene transcription factor C/EBP homologous protein (CHOP, also called DDIT3 or GADD153) for the cells subjected to the above experiments. Upregulation of GRP78/BiP mRNA was observed in the LG-, HG-LG-, and Tg-treated groups, and this observation was confirmed in the Western blotting results obtained 24 h posttreatment (Figure 1).

### Enhancement of RuV Infection and Replication in First-Trimester Trophoblast Cells Under Low-Glucose Stress Conditions

#### Enhanced Intracellular Localization of RuV Capsid Protein in LG-Stressed Cells by Immunofluorescence Assay

**Figure 2:**
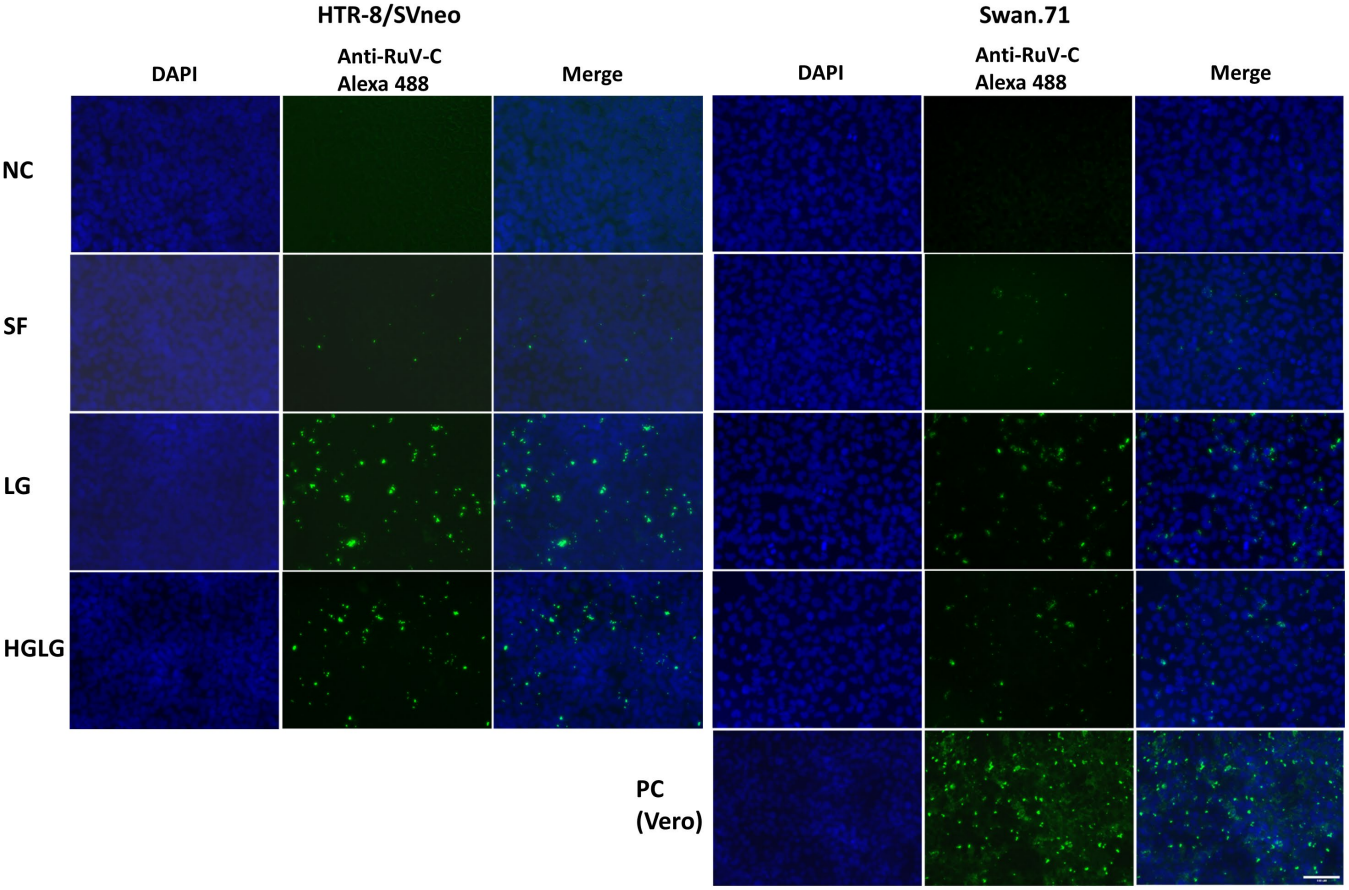
Microscopy images of HTR-8/SVneo and Swan.71 cells infected with RuV under LG stress conditions. The cells were seeded onto 6-well plates containing glass coverslips and cultured for 1 to 2 days before LG stress treatments and viral infection. The cells were fixed 48 hpi and labeled with a mouse monoclonal anti-rubella viral capsid antibody (ab34749, Abcam), followed by an Alexa 488-conjugated goat anti-mouse IgG (H + L) secondary antibody (green). Nuclei were stained with DAPI (blue). RuV-infected Vero cells were used as positive controls. Trophoblast cells mock-infected, incubated with heat-inactivated RuV, or stained with mouse serum were used as negative controls. Images are representative of 3 independent experiments. RuV-C, rubella virus capsid. Scale bar: 100 μM.

The studied trophoblast cells were cultured under LG stress conditions prior to RuV infection. An immunofluorescence assay using an anti-capsid protein antibody showed an enhancement of the intracellular localization of RuV capsid protein in the LG-stressed cells compared with the control (Figure 2).

#### High Percentages of RuV-Positive Cells Under LG Stress Conditions as Shown by Flow Cytometric (FCM) Analysis

**Figure 3:**
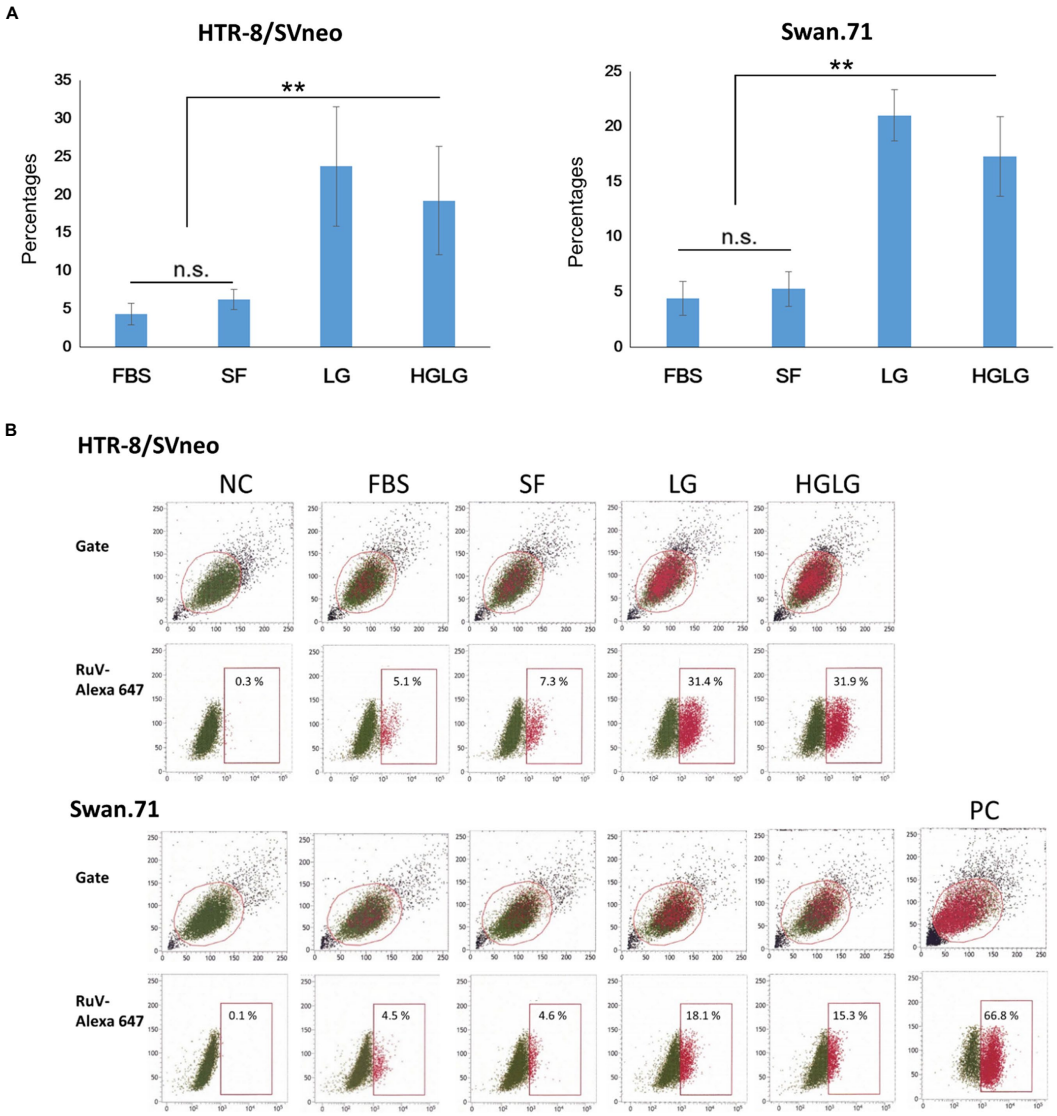
**(A)** Percentages of the studied trophoblast, HTR-8/SVneo and Swan.71 cells positive for RuV as determined by FCM analysis at 24 hpi. Trophoblast cells were seeded at a density of 5 × 10^3^ cells/well in a 96-well plate. After LG stress treatments and RuV infection at an MOI of 5–10, the cells were collected at 24 to 48 hpi and labeled with a Live/Dead Fixable Near-IR Dead Cell Stain Kit to exclude dead cells from analysis. Intracellular staining was performed with a mouse monoclonal anti-rubella viral capsid antibody followed by an Alexa-488 conjugated goat anti-mouse IgG (H + L) secondary antibody. Mock-infected cells using culture medium were used as negative controls (NCs). Vero cells infected with RuV performed in parallel were used as positive controls (PCs). The results are expressed as the mean of at least triplicate experiments in each group, and each graph is representative of three independent experiments. ^**^p < 0.01. **(B)** Representative images of flow cytometry analysis. Numbers displayed inside each panel correspond to the percentage of the cells positive for RuV capsid protein of the parent gated population.

For the cells cultured in SF medium used as the control for LG stress experiments, an average of 5% (ranging from 3 to 7%) of the gated cells showed signals of infection. However, noticeably high percentages of RuV-positive cells were observed in the LG- and HGLG-treated groups, ranging from 15 to 33%, as noted in several repeated experiments (Figure 3). No significant difference was noted in the percentages of RuV-positive cells of the cells cultured in complete medium (FBS) compared with those cultured in the control, which is consistent with no difference in the expression of GRP78 and CHOP between these two groups (Figures 1, 3).

#### Successful Viral Progeny Production in LG-Stressed Trophoblast Cells

**Figure 4:**
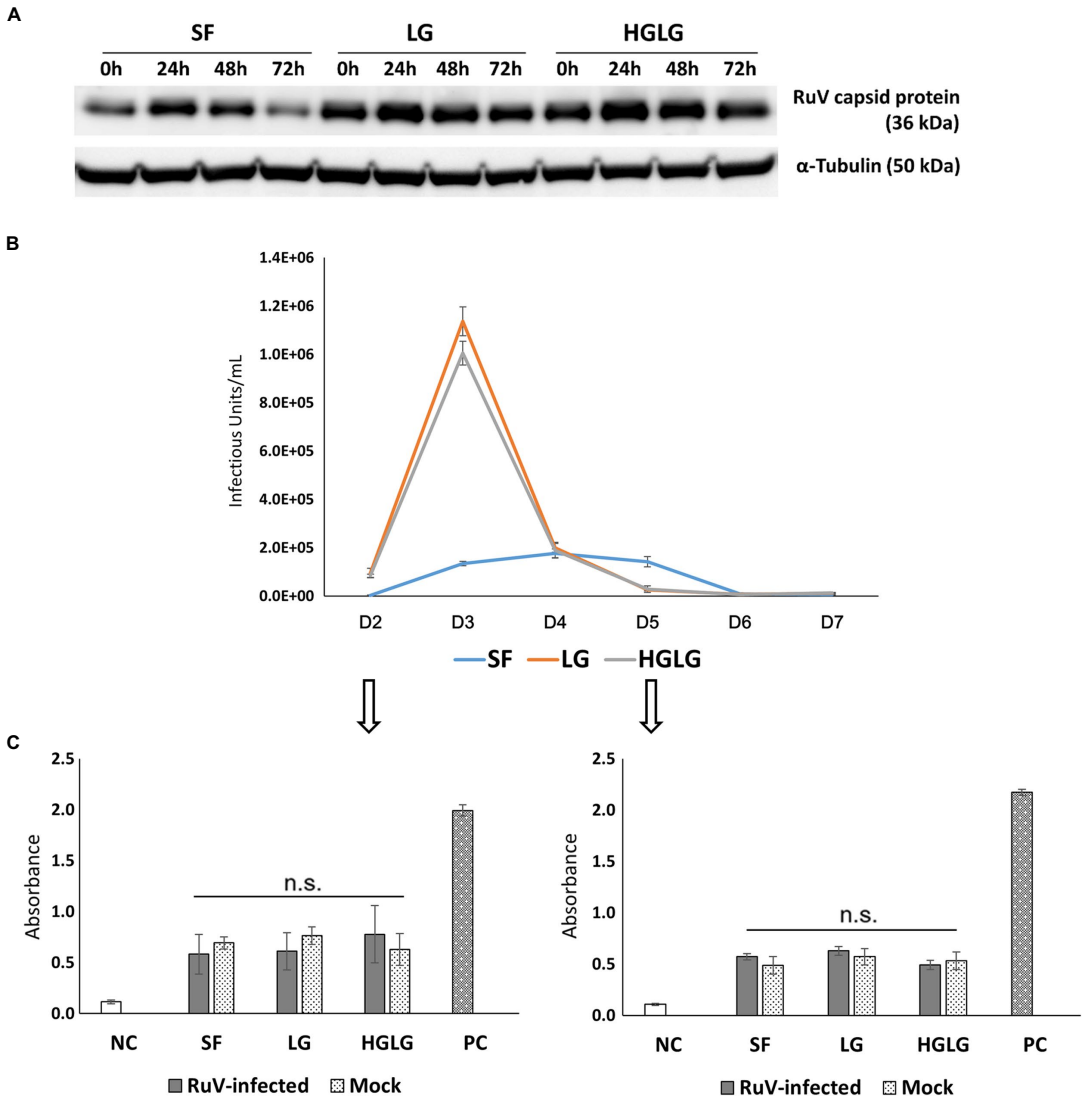
Investigation of viral replication and apoptosis in LG-stressed RuV-infected HTR-8/SVneo cells. **(A)** Viral replication in LG-stressed RuV-infected cells shown by Western blotting. Trophoblast cells were cultured in 12-well plates, subjected to LG stress, and then inoculated with RuV. At 0, 24, 48, and 72 hpi, the lysate was collected for Western blot analyses. Cells cultured in SF medium were used as the controls for LG stress treatments. **(B)** Titers of viral progeny in the supernatants collected from day 2 to day 7 pi as determined by FCM analysis. Trophoblast cells were cultured in a 96-well plate and subjected to LG stress and RuV infection as described. From 48 hpi, the entire supernatant was collected daily and replaced with a new medium until day 7 pi. The collected supernatants were used to infect freshly prepared Vero cells and titered by FCM analysis as described elsewhere. The graph is representative of two independent experiments. The results are expressed as the mean of at least triplicate experiments in each group, and the graph is representative of two independent experiments. **(C)** No enhancement of apoptosis of LG stress-infected trophoblast cells. Trophoblast cells were seeded at a density of 5 × 10^3^ cells/well onto a 96-well flat-bottom plate as specified by the manufacturer. After 2 d of culture and subsequent LG stress and RuV infection, ELISA detection of single-stranded DNA present in apoptotic cells was performed at days 2 and 5 pi. The cells subjected to LG stress and mock infection were treated in parallel and used for comparison. Single-stranded DNA provided in the ELISA kit was used as a positive control. The results are expressed as the mean of at least triplicate experiments in each group, and the graph is representative of two independent experiments. NC, negative control; mock, mock-infected with virus-free medium; PC, positive control; n.s., nonsignificant.

Successful replication of RuV in LG-stressed cells was observed with Western blot analysis at 24 hpi (Figure 4A). Viral titer monitoring using FCM analysis showed substantial viral progeny production from day 2 to day 5 pi. The highest viral titers of the supernatants of the cells precultured in LG or HGLG media were observed on day 3 pi (Figure 4B).

#### No Difference in Apoptosis Between Uninfected and RuV-Infected LG-Stressed Cells

Apoptosis assays were performed on trophoblast cells grown in 96-well plates on days 2 and 5 pi. The cells without RuV infection but simultaneously subjected to LG stress conditions were also subjected to the apoptosis assay. No significant difference in apoptosis was found between the LG-stressed and non-LG-stressed groups in the RuV-infected experimental groups. The absorbance values of LG-stressed cells infected with RuV were not different from those of the control cells or the stressed mock-infected cells (incubated with a culture medium or heat-inactivated virus). These results suggest that no significant apoptosis occurred in either of the RuV-infected groups (mock or LG-stressed) until day 5 pi (Figure 4C).

### Enhancement of RuV Binding to LG-Stressed Trophoblast Cells by the Viral Binding Assay

**Figure 5:**
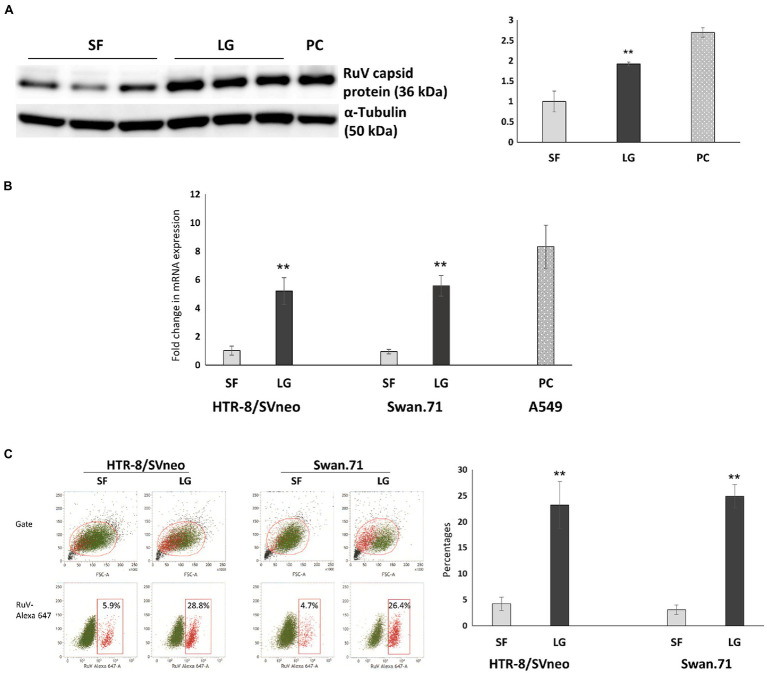
Viral binding assays showed an enhancement of RuV binding to LG-stressed trophoblast cells. After LG stress treatment, the cells were inoculated with RuV at an MOI of 5 to 10 on ice for 1 h. After washing, the cells were lysed and subjected to Western blot analysis, RNA extraction, and real-time PCR, or surface staining procedures for RuV were performed, and the cells were analyzed by FCM. **(A)** Increased expression of the RuV capsid protein in HTR-8/SVneo cells under LG stress, as shown by Western blot analysis. The graph was constructed based on band intensities (arbitrary units) normalized to the value of the cells cultured in SF medium. Values shown are the mean of three independent experiments ± SEM. PC, A549 cells. **(B)** Increased viral RNA expression in HTR-8/SVneo and Swan.71 cells cultured in LG medium. Data shown are the means of fold-change of relative expression values (normalized to the internal control PPIA gene expression) ± SEM. **(C)** Increased percentages of RuV-positive HTR-8/SVneo and Swan.71 cells cultured in LG medium determined by FCM analysis. **(B,C)** The results are expressed as the mean (± SEM) of at least triplicate experiments in each group, and the graph is representative of two independent experiments. ^**^p < 0.01.

After LG stress treatment, the cells were inoculated with RuV on ice for 1 h. The cells were washed and subjected to surface staining procedures for RuV. RNA was extracted for real-time PCR, and lysates were collected for Western blot analysis. The results obtained by FCM analysis showed that the percentages of RuV-positive cells increased significantly up to 5-fold in LG-stressed cells compared to the control cells (Figure 5C). These findings were confirmed by real-time PCR, which showed a corresponding increase in RuV RNA expression after normalization to the internal control, PPIA gene expression (Figure 5B). The enhancement of RuV binding to LG-stressed trophoblast cells was also observed with Western blot analysis (Figure 5A).

## Discussion

In this study, under the experimental LG stress conditions using LG or HGLG media, an enhancement of RuV infection of the human first-trimester trophoblast cell lines was noted. Using an MOI of 5 to 10 to ensure that every single cell had the chance to theoretically come in contact with at least 1 infectious virus, the percentages of RuV-positive cells of the LG-stress groups reached over 30% on FCM analysis (Figures 2, 3). Increases in the percentages of RuV-infected cells observed along with enhanced expression of ER stress markers (GRP78 and CHOP) imply that LG-induced ER stress could enhance RuV infection in these trophoblast cells.

There is a gap in our understanding of the mechanisms of transplacental RuV infection in early pregnancy. Well-established studies of CRS found that this congenital abnormality often occurred in early pregnancy, with an incidence as high as 90% (Hobman, 2013). Current articles have reported that trophoblast cells are resistant to many viral infections, including RuV (Bayer et al., 2015; Trinh et al., 2018). Our latest experimental results showed that RuV has low infectivity to the first-trimester trophoblast cell lines HTR-8/SVneo and Swan.71 *in vitro* (Pham et al., 2022). Early studies suggested that transplacental RuV infection targets fetal endothelial cells and Hofbauer cells instead of trophoblasts (Perelygina et al., 2013, 2015). However, a very early study reported an *in vitro* RuV infection model employing choriocarcinoma cell lines and suggested some conditions permitting transplacental infection *via* trophoblasts (Sugiura, 1982). Thus, we investigated possible *in vivo* conditions that enable RuV infection into trophoblasts. Subsequently, with the finding of an increase in the susceptibility of these cells to RuV under low-glucose stress conditions, the present study provides a promising approach for understanding the mechanisms of RuV infection of first-trimester trophoblast cells.

Not only were significant increases in RuV infection in LG-induced ER-stressed trophoblast cells noted, but successful replication and a release of progeny virus into the supernatant were also observed (Figures 4A,B). Substantial viral progeny production was observed from day 2 to day 5 pi, with a peak at day 3 pi. These findings suggest that RuV can infect EVTs during pregnancy under some LG-induced ER stress conditions, replicate in these cells, and successfully produce progeny virus, which leads to RuV infection of other fetal cells and causes unfavorable outcomes for the fetus. To support the study findings, future studies utilizing other appropriate cells, such as primary trophoblast cells and explant culture, as well as expanding to other trophoblast types, such as BeWo cells, are necessary.

RuV is well known for its ability to suppress apoptosis *via* its capsid protein (Willows et al., 2014). No significant amount of apoptosis occurred in RuV-infected, ER-stressed cells on days 2 and 5 pi in the present study, which suggests that the viral progeny production pattern was not associated with apoptosis, but that an intracellular resource was necessary for viral production.

Recent findings have shown that RuV has two distinct binding mechanisms for viral entry: a Ca^2+^-dependent mechanism observed in lymphoid cells and a Ca^2+^-independent mechanism involving unidentified RuV receptor(s) (Otsuki et al., 2018). Myelin oligodendrocyte glycoprotein (MOG) is a receptor for RuV, but it is primarily expressed in the central nervous system (Cong et al., 2011) and not detected in trophoblasts (Trinh et al., 2018). We used the viral binding assay and observed successful RuV binding of the studied trophoblast cells under LG culture, which is representative of the experimental LG-induced ER stress treatment. Compared to the controls, the treatment increased viral binding to the studied cells up to 5-fold. The finding implies that alternative receptors expressed during the LG-induced ER stress process may be involved in viral entry. Recent studies suggest that microorganisms can utilize proteins or components of the cellular stress responses to facilitate their infection process. Viruses, including the SARS-CoV-2, can bind to the GRP78 protein involved in the unfolded protein responses in ER stress for their entry into target cells (Rayner et al., 2020; Gonzalez-Gronow et al., 2021; Trinh, 2022). Therefore, to determine which proteins induce RuV infection enhancement in the treated trophoblast cells in this study, the GRP78 and other proteins such as GRP94 of the low-glucose ER stress response are highly potential candidates.

In conclusion, although the mechanisms underlying RuV entry into LG-induced ER-stressed cells and RuV replication in these cells are not known and require further investigation using appropriate approaches, these findings suggest that a certain degree of LG-induced ER stress in early pregnancy may facilitate infection and cause CRS.

## Data Availability Statement

The original contributions presented in the study are included in the article/supplementary material; further inquiries can be directed to the corresponding authors.

## Author Contributions

QT, SK-A, HU, and SH: conceptualization. QT, KT, NP, and SK-A: methodology and formal analysis. SK-A and SH: validation. QT, KT, NP, and TN: investigation. KT, CT, SO, HU, SK-A, and SH: resources. QT and NP: writing—original draft preparation. QT, KT, CT, TN, SO, SK-A, HU, and SH: writing—review and editing. QT and SH: funding acquisition. All authors contributed to the article and approved the submitted version.

## Funding

This study was supported by Grants-in-Aid for Scientific Research under the Japan Society for the Promotion of Science (JSPS KAKENHI) grant numbers 17H04341 (to SH) and 20 K08829 (to QT).

## Conflict of Interest

The authors declare that the research was conducted in the absence of any commercial or financial relationships that could be construed as a potential conflict of interest.

## Publisher’s Note

All claims expressed in this article are solely those of the authors and do not necessarily represent those of their affiliated organizations, or those of the publisher, the editors and the reviewers. Any product that may be evaluated in this article, or claim that may be made by its manufacturer, is not guaranteed or endorsed by the publisher.
